# Survival in patients diagnosed with melanoma in situ compared to the general population. A Swedish population-based matched cohort study

**DOI:** 10.1016/j.eclinm.2023.102284

**Published:** 2023-10-24

**Authors:** Ylva Naeser, Rasmus Mikiver, Christian Ingvar, Mats Lambe, Gustav J. Ullenhag

**Affiliations:** a Department of Oncology, Uppsala University Hospital, Entrance 101, 751 85, Uppsala, Sweden; b Department of Immunology, Genetics and Pathology, Uppsala University, Dag Hammarskjölds väg 20, 751 85, Uppsala, Sweden; c Regional Cancer Center South-East, Kungsgatan 23, 582 18, Linköping, Sweden; d Department of Clinical and Experimental Medicine, Linköping University, 581 83, Linköping, Sweden; e Department of Clinical Sciences, Surgery, Lund University, 221 84, Lund, Sweden; f Department of Medical Epidemiology and Biostatistics, Karolinska Institutet, 171 77, Stockholm, Sweden; g Regional Cancer Center Central Sweden, 751 85, Uppsala, Sweden

**Keywords:** Melanoma in situ, Prognosis, Survival, Socioeconomic status, Comorbidity

## Abstract

**Background:**

The incidence of melanoma in situ (MIS) is increasing even more rapidly than the incidence of cutaneous malignant melanoma (CMM). No previous studies have in detail investigated the survival in individuals diagnosed with MIS compared to the general population.

**Methods:**

This population-based study included individuals with MIS diagnosed in Sweden between 2001 and 2010 and randomly selected MIS-free comparators matched on age, sex and county of residence. Exclusion criterion was a previous CMM. Data on socioeconomic status (SES) including educational level, income and marital status, comorbidity and cause of death were obtained from population-based registers. Overall survival (OS) was estimated by the Kaplan–Meier method. The mortality risk adjusted for SES and comorbidity was assessed by multivariable Cox regression analyses.

**Findings:**

The survival analyses included 7963 cases and 39,662 comparators. Median age at MIS diagnosis were 63 (IQR 50–75) and 67 (IQR 57–76) years in women and men respectively. Median follow-up time was 120 months (IQR 102–152 months). In individuals with MIS, the ten-year OS was 77% (95% CI 0.76–0.78) compared to 72% (95% CI 0.72–0.73) in comparators. The MIS patients had a higher SES and lower comorbidity burden than the comparators. In a fully adjusted multivariable analysis, including 7772 cases and 38,103 comparators, the mortality was significantly lower in women with MIS (HR 0.88, 95% CI 0.82–0.94) compared to the background population. The corresponding estimate in men was HR 0.94 (95% CI 0.88–1.0). The risk of melanoma-related deaths during the study period was ten-fold higher in MIS patients.

**Interpretation:**

Despite being at increased risk of developing CMM, MIS patients had a better OS compared to their matched comparators from the background population, findings which could not fully be explained by differences in SES and comorbidity. Our results are reassuring and should be communicated to patients who have been diagnosed with MIS.

**Funding:**

Stiftelsen Onkologiska Klinikens i Uppsala Forskningsfond, Mats and Stefan Paulsson Trust, Medicon Village, Lund and 10.13039/501100005423Uppsala University Hospital (ALF).

**Research in context**

**Evidence before this study**

The incidence of melanoma in situ (MIS) is increasing even more rapidly than the incidence of cutaneous malignant melanoma (CMM). Socioeconomic status (SES) is associated with the incidence and outcome of CMM. We searched PubMed for publications until July 1, 2023, using the search terms “melanoma in situ” AND “survival” and “melanoma” AND “socioeconomic status” and “melanoma” AND “socioeconomic factors”. Most studies assessing possible associations between SES and melanoma incidence have been restricted to CMM diagnoses. However, one Canadian study has reported a positive association between high income and the risk of MIS. One large U.S. study reported that the relative 5-year overall survival (OS) in MIS patients was similar to that in the general population. In an English report, the net 5-year OS for MIS patients was higher than the expected OS in the general population. Also, a recently published U.S. study found that patients with a history of a MIS diagnosis were living for up to 15 years longer compared to age-, sex-, race-, ethnicity-matched individuals in the general population.

**Added value of this study**

No previous studies have investigated the survival in MIS patients compared to matched comparators representing the general population. In this large, population-based study we compared the OS in MIS patients and matched comparators free of MIS. We found that both men and women with MIS had a significantly better OS up to at least ten years after diagnosis. Comorbidity burden was lower and SES higher among individuals diagnosed with MIS. Adjustment for these factors could not fully explain a lower risk of mortality in MIS patients.

**Implications of all the available evidence**

We believe that our results should impact the information provided to MIS patients given that on a group-level their life-expectancy is better than in the general population.

## Introduction

The incidence of Melanoma in situ (MIS), a precursor stage of cutaneous malignant melanoma (CMM) is rising even more rapidly than the incidence of CMM.^1^^,^^2^ While MIS is not associated with the risk of metastatic spread, individuals with MIS are at an increased risk of developing CMM compared to the general population.^3^, ^4^, ^5^ Few studies to date have investigated the long-term outcomes in MIS patients. Results from a register-based U.S. study indicated that the life expectancy in individuals with MIS is similar to that of the general population.^5^ In a report including all registered skin cancers in England between 2013 and 2019, the highest five-year relative OS survival was found in MIS patients. The MIS patients' relative five-year OS was higher than that in the background population.^6^ Also, a recently published US study found that patients with a history of a MIS diagnosis were living for up to 15 years longer compared to age-, sex-, race-, ethnicity-matched individuals in the general population.^7^

While rates of thick CMMs have increased, the overall increase in the incidence of CMMs is primarily driven by thin melanomas.^1^ This might reflect an increased awareness of the risk of developing CMM and a tendency to seek early advice for skin abnormalities. While men have a higher risk of developing CMM compared to women,^8^ there is no clear sex difference in the incidence of MIS.^9^

Women are more often diagnosed with thinner CMMs and hence have a better prognosis than men.^10^ In Sweden, the majority of CMMs are thin, below or equal to one mm in thickness. Based on data in the SweMR between 2020 and 2021, the proportion of thin CMMs was higher in women (61%) as compared to men (57%).^9^ In addition, results from several studies indicate that stage-specific survival is better in women.^10^, ^11^, ^12^, ^13^ Although data on survival differences between men and women with MIS are scarce, one study including patient with MIS in the head and neck region found a better OS in women.^14^

The influence of SES on both the incidence and prognosis of CMM is well established; studies from the mid 80′s and onward have reported a higher incidence of CMM, thinner tumors and better prognosis in groups with high SES.^15^, ^16^, ^17^ Likely reasons for these findings include differences in health care seeking behaviors and lifestyle, including exposure to ultraviolet radiation associated with travel to sunny destinations. Most studies to date have examined the role of SES in relation to CMM incidence. To the best of our knowledge, only one study to date has assessed the association between SES and the risk of MIS and found a significantly higher rate of MIS in high-compared to low-income groups in Canada.^18^ One study restricted to MIS of the vulva found a significantly better OS in women with high SES.^19^

The aim of this study was two-fold. First, to investigate whether OS differs between patients with MIS and a matched comparison cohort with individuals free of MIS and if any such difference can be explained by socioeconomic factors and comorbidity burden. Second, to examine and compare the distribution of comorbid conditions.

## Methods

### Data sources and data collection

The SweMR is a quality register to which clinical data is reported with a completeness exceeding 98% compared to the Swedish Cancer Register (SCR) to which reporting is mandated by law.^9^ MIS was reported to the Swedish Melanoma Register (SweMR) between 1990 and 2010, and thereafter only to regional cancer registers. Since not all regions continued to report MIS the registration of MIS is incomplete after year 2010. Hence, we chose not to include MIS patients diagnosed later than 2010 in this study.

For the purpose of the present matched cohort study, we used data available in the research database MMBaSe that was generated by individual-level record linkage between the SweMR, the National Patient Register (NPR), the Cause of Death Register (CDR), the SCR and the Longitudinal Integrated Database for Health Insurance and Labor Market Studies (LISA). Cases were defined as individuals with a diagnosis of MIS as first registered diagnosis in the SweMR between 1996 and 2011. A comparison cohort was established from the Population Register (PR) by random selection of up to five MIS free individuals (comparators) per case who were matched on age, sex and county of residence at the time of the diagnosis of the corresponding case. For both cases and comparators, data on SES, comorbidity and deaths were retrieved.

Comorbidity data was obtained from the NPR which contains data on hospital admission and discharges codes according to International Classification of Diseases (ICD) from all Swedish hospitals since 1987. Beginning in 2001, the register also includes information on hospital out-patient visits.^20^ Information on date and underlying cause of death was retrieved from the CDR based on to the international version of ICD-10.^21^

Data on income, marital status and educational level were obtained from LISA, a nationwide continuously updated database including individuals 16 years and older.^22^

By use of the Swedish personal identity number assigned to all residents, linkage rates are very high. Percentage of non-linkage was lower than 2% across registers. The proportion of missing data was less than 2% for the variables educational level and income and less than 1% for marital status. The data quality in Swedish population-based registers is generally high and are being extensively used in epidemiological studies.^23^, ^24^, ^25^

### Socioeconomic status

For the purpose of the present study, three socioeconomic indicators were used: highest achieved educational level, income and marital status. Educational level was categorized into three groups based on number of years of schooling: low ≤9 years, middle 10–12 years and high ≥13 years, corresponding to mandatory school, high school and post-high school (college or university). Income data was retrieved as family annual disposable income and assessed in relation to an income above or below the median for all study participants. Marital status was divided into four major groups: married, unmarried, divorced or widower. Socioeconomic indicators for both cases and comparators were assessed at the date of diagnosis of the case (index date).

### Comorbidity

The Charlson Comorbidity Index (CCI), originally published in 1987 to predict 1-year mortality,^26^ remains a widely used method to estimate comorbidity burden. The CCI is based on a list of medical conditions where each diagnosis contributes a specified point based on severity and then summarized to a total score. The original CCI has been updated and revised by several authors. We used the CCI algorithm published in 2021 based on the updated Charlson/Quan index^27^^,^^28^ and adapted for register-based research in Sweden.^29^

The CCI was categorized into three groups no (CCI 0), mild (CCI 1) and severe (CCI +2). For cases, all diagnoses except CMM (ICD C43), until 14 days before the diagnosis of the index diagnosis of MIS was included in the CCI. For comparators, all diagnoses until the index date were included.

### Study population

Inclusion criteria for the cases was a diagnosis of MIS registered in SweMR between January 1, 2001 and December 31, 2010 without a previous diagnosis of CMM. All melanoma in situ including lentigo maligna (LM) at all sites were included (Table 1A, appendix page 1).

If more than one diagnosis of MIS was identified, the first was selected. Exclusion criteria included a diagnosis of CMM reported in the SCR, but not recorded in SweMR before the index date, individuals where the personal identifier might have been reused or duplicated or age below 18 years at date of diagnosis. If no comparators were available, the case was excluded.

**Fig. 1:**
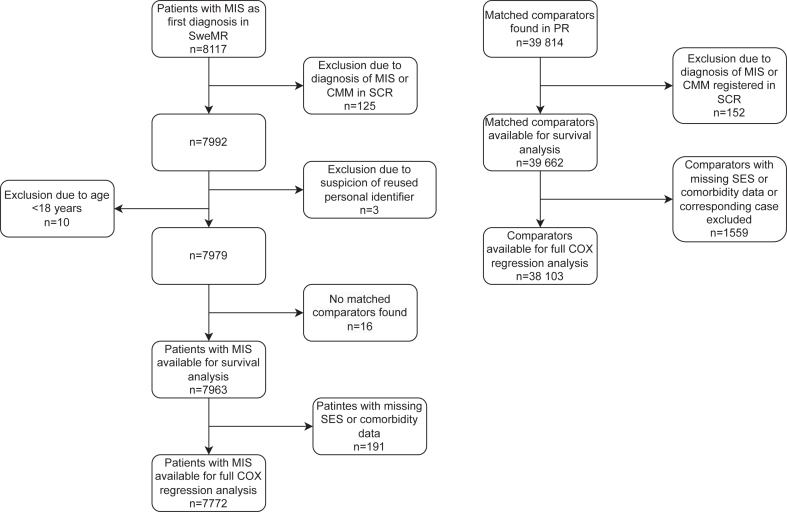
Final study population for analysis of survival and Cox regression analysis and reasons for exclusion for cases and matched comparators. Abbreviations used: Melanoma in situ (MIS), the Swedish Melanoma Register (SweMR), Cutaneous Malignant Melanoma (CMM), the Swedish Cancer Register (SCR), Socioeconomic Status (SES) and the Population Register (PR).

We identified 8117 patients registered in SweMR with MIS as first diagnosis, without a previous CMM diagnosis. Following cross-check against the SCR, 125 cases were excluded due to a diagnosis of CMM before the MIS diagnosis which was not recorded in SweMR. Another 29 cases were excluded due to the following reasons: Suspicion of reused personal identifier (n = 3), no available comparators (n = 16) and age below 18 years (n = 10). In this way, 7963 cases MIS cases without a previous diagnosis of CMM were available for the survival analyses. The number of matched comparators from the PR was 39,814, but after cross-check against the SCR, 152 individuals were excluded due to a diagnosis of CMM and/or MIS before the index date, yielding 39,662 comparators available for analyses (Fig. 1).

The full multivariable Cox regression analysis for estimation of risk of death during the study period encompassed 7772 cases and 38,103 matched comparators with all SES and comorbidity variables available.

### Ethical approval

Ethical approval for the construction of MMBaSe and associated study projects was granted by the Regional Ethics Board in Uppsala (# 2018/405). The project was also approved by the Regional Cancer Center South-East which is the register holder for SweMR and owner of MMBaSe. The research data in MMBaSe were made available in an anonymized format.

### Statistical analysis

Descriptive statistics were used to characterize cases and comparators at the date of diagnosis of the case (index date). Categorical variables were presented as numbers and percentages. Income was presented as mean with standard deviation and age as median with interquartile range. Descriptive statistics were compared with the Chi-square test for categorical variables and the Mann–Whitney U-test for continuous variables. The level of significance was 0.05 and all p-values were two-tailed.

Overall survival estimates and 95% confidence intervals (CIs) were assessed by the Kaplan–Meier method. Cox regression analyses were applied to compare controls with comparators with results presented as hazard ratios (HRs) with 95% CIs. Multivariable analyses were performed with stepwise adjustment for educational level, income and comorbidity separately and in a full model with adjustment for all factors. Cases and comparators with missing variables were excluded. If a case was excluded, the corresponding matched comparators were also excluded from the analysis. Separate analyses were performed in men and women. All statistical analyses were performed using R Statistical Software (v4.0.3 R Core Team 2020).

### Role of the funding source

The funders had no role in study design, data collection, data analysis, data interpretation or writing of the report. All authors had access to the dataset and final responsibility for the decision to submit for publication.

## Results

### Demographic and socioeconomic characteristics

The majority of MIS cases were women (55% [4406]) with a median age at diagnosis of 63 years (IQR 50–75) compared to 67 (IQR 57–76) years in men. Individuals with MIS had a significantly higher educational level and disposable income than the comparators. Median income for comparators was 133,300 (IQR 102,500–186,700) Swedish crowns (SEK) and 150,450 (IQR 114,300–211,200) SEK for cases. Also, cases were significantly more often married and less often divorced (Table 1).

**Table 1:** Demographic, clinical and socioeconomic characteristics of patients with melanoma in situ (cases) and matched comparators.

Parameter	Cases n = 7963 (%)	Comparators n = 39,662 (%)	p-value
Sex			0.99
Male	3557 (45)	17,711 (45)	
Female	4406 (55)	21,951 (55)	
Age, median (IQR)	65 (53–76)	65 (53–76)	0.88
Age years			1.0
18–39	758 (9.5)	3788 (9.6)	
40–59	2171 (27)	10,829 (27)	
60–69	1961 (25)	9769 (25)	
70–79	1809 (23)	8996 (23)	
80+	1264 (16)	6280 (16)	
Marital status			<0.001
Married	4657 (59)	20,393 (51)	
Divorced	1005 (13)	6221 (16)	
Unmarried	1196 (15)	6939 (18)	
Widower	1039 (13)	6086 (15)	
Missing	66 (0.83)	23 (0.058)	
Educational level			<0.001
Low	4154 (52)	24,801 (63)	
Middle	2053 (26)	8696 (22)	
High	1610 (20)	5452 (14)	
Missing	146 (1.8)	713 (1.8)	
Disposable income, mean (family)	1842 (1657)	1611 (1458)	<0.001
Missing	107 (1.3)	0 (0.0)	
Charlson Comorbidity Index			0.020
0	6195 (78)	30,822 (78)	
1	1393 (17)	6691 (17)	
+2	375 (4.7)	2149 (5.4)	

Number of participants (%) unless otherwise specified. IQR, interquartile range; disposable income per consumption unit in ×100 Swedish crowns (SEK).

### Comorbidity

At the time of diagnosis, the comorbidity burden was significantly lower in cases with a lower prevalence for six out of eighteen diagnosis groups included in the CCI. This included cerebrovascular disease, the second most common comorbidity in both groups. However, the rate of malignancies was significantly lower in the comparison group, the most common concomitant condition in both cases and comparators. The rate of a metastatic cancer was similar in both groups (Table 2). The rate of a subsequent CMM in cases was 5.7% (450) and 0.8% (319) in the comparison group.

**Table 2:** Charlson Comorbidity Index (CCI) in patients with melanoma in situ (cases) and matched comparators.

	Cases n (%)	Comparators n (%)	p-value
Charlson Comorbidity Index (CCI)			0.020
0	6195 (78)	30,822 (78)	
1	1393 (17)	6691 (17)	
2+	375 (4.7)	2149 (5.4)	
Congestive heart failure	157 (2.0)	946 (2.4)	0.028
Peripheral vascular disese	113 (1.4)	675 (1.7)	0.079
Cerebrovascular disease	356 (4.5)	2245 (5.7)	<0.001
Chronic obstructive pulmonary disease	65 (0.82)	508 (1.3)	0.001
Chronic other pulmonary disease	102 (1.3)	483 (1.2)	0.68
Rheumatic disease	162 (2.0)	685 (1.7)	0.065
Dementia	30 (0.38)	345 (0.87)	<0.001
Hemiplegia	6 (0.075)	55 (0.14)	0.20
Diabetes without chronic complication	8 (0.10)	30 (0.076)	0.62
Diabetes with chronic complication	96 (1.2)	712 (1.8)	<0.001
Renal disease	47 (0.59)	224 (0.56)	0.85
Mild liver disease	16 (0.20)	125 (0.32)	0.11
Liver special	0 (0.0)	5 (0.013)	0.60
Severe liver disease	0 (0.0)	26 (0.066)	0.015
Peptic ulcer disease	78 (1.0)	448 (1.1)	0.27
Malignancy	719 (9.0)	2515 (6.3)	<0.001
Metastatic solid cancer	19 (0.24)	132 (0.33)	0.21
Aids	1 (0.013)	7 (0.018)	1.0

### Overall survival

Median duration of follow-up was 120 months (IQR 98–152); 123 months for the cases (IQR 102–154) and 120 months for the comparators (IQR 97–152). A total of 2264 individuals (28%) in the MIS patient group and 12,824 individuals (32%) in the matched cohort died during the study period.

#### Men and women combined

**Fig. 2:**
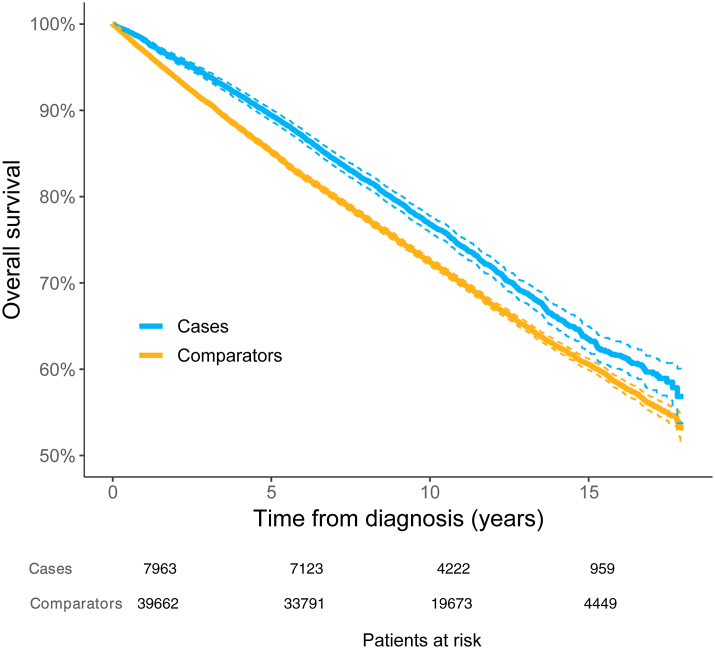
Overall survival: Patients with melanoma in situ (MIS cases) and matched comparators. Dashed lines represent 95% CI.

Patients with MIS had a significantly better OS with 90% (95% CI 0.89–0.90) alive at five years compared to 85% (95% CI 0.85–0.86) of the comparators. The corresponding estimates at ten years were 77% (95% CI 0.76–0.78) and 72% (95% CI 0.72–0.73), respectively. After fifteen years, a significant difference remained with 63% (95% CI 0.62–0.65) of cases alive compared to 61% (95% CI 0.60–0.61) of the comparators (Fig. 2).

#### Men

**Fig. 3:**
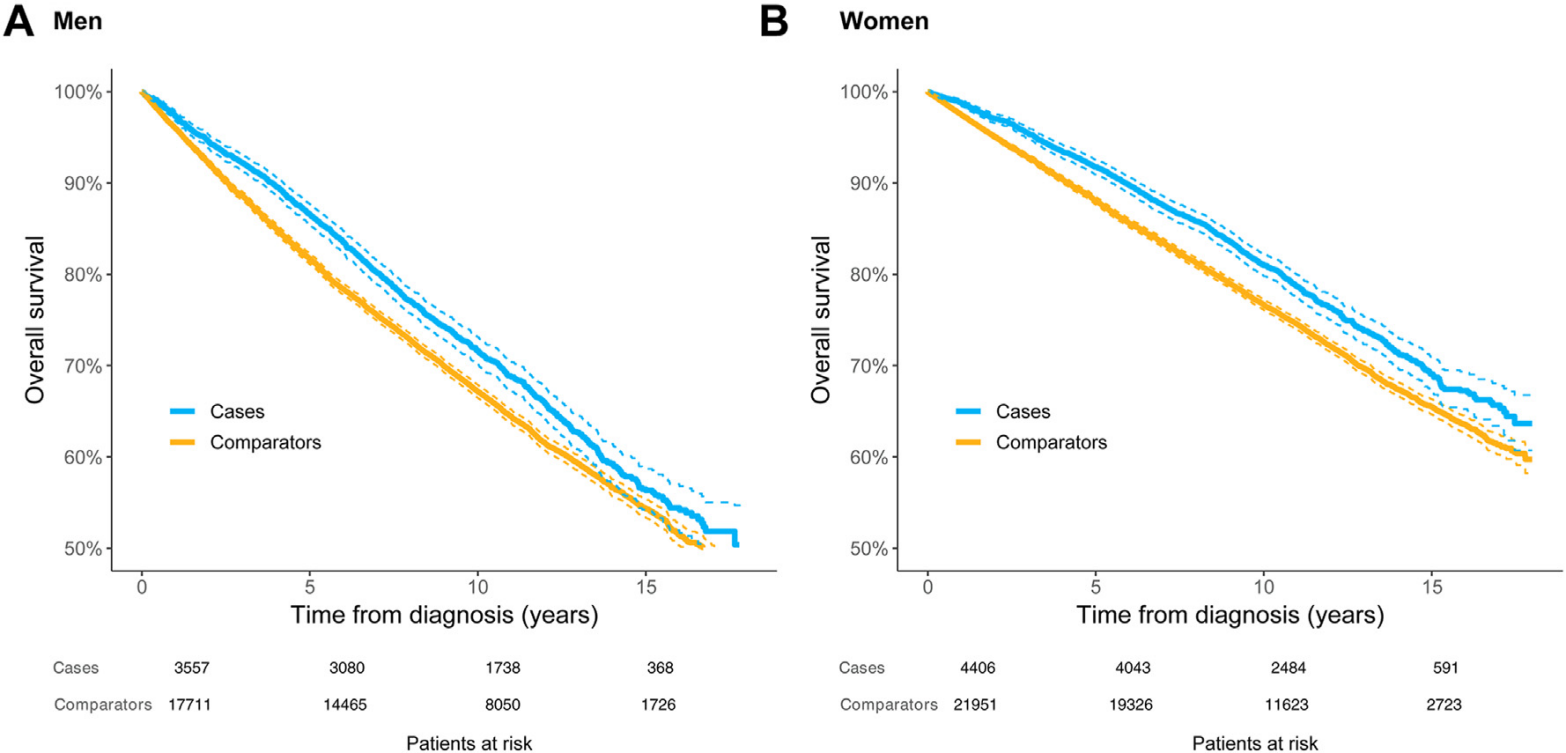
A. Overall survival: Male patients with melanoma in situ (MIS cases) and matched comparators. Dashed lines represent 95% CI. B. Overall survival: Female patients with melanoma in situ (MIS cases) and matched comparators. Dashed lines represent 95% CI.

In men, there were differences in OS between MIS patients and comparators at five years with 87% (95% CI 0.86–0.88) of cases alive vs 82% (95% CI 0.81–0.82) of the comparators. At ten years, a significant difference remained; 72% (95% CI 0.70–0.73) vs 67% (95% CI 0.67–0.68). At fifteen years, there was no statistically significant difference in OS; 56% (95% CI 0.54–0.59) and 54% (95% CI 0.53–0.55) respectively were alive in each group (Fig. 3A).

#### Women

In women, the OS in the MIS patient group was significantly better than in the matched cohort at five, ten and fifteen years. At five years 92% (95% CI 0.91–0.93) of the cases were alive vs 88% (95% CI 0.88–0.89) of the comparators. At ten and at fifteen years these differences were 81% (95% CI 0.80–0.82) vs 77% (95% CI 0.76–0.77) and 69% (95% CI 0.67–0.71) vs 66% (95% CI 0.65–0.66), respectively (Fig. 3B).

### Mortality

#### Univariable and multivariable analysis

In univariable Cox proportional hazard regression analysis, the risk of overall mortality was lower in the MIS patient group (Hazard Ratio (HR) 0.84, 95% CI 0.80–0.88) compared to the matched cohort. This difference was more pronounced in women (HR 0.82, 95% CI 0.76–0.87) than in men (HR 0.86, 95% CI 0.81–0.91).

In stepwise adjustments, both a high income and high educational level were associated with a lower risk of death and attenuated the risk estimates in the univariable analyses (Tables A2 and A3; appendix page 1 and 2). A high comorbidity burden was associated with an increased mortality, but did only marginally affect the estimate in the univariable analysis (Table A4; appendix page 2).

In a fully adjusted model including educational level, income, marital status and comorbidity (CCI), the risk of death remained significantly lower in the MIS patient group compared to the matched cohort (HR 0.90, 95% CI 0.86–0.94). A borderline significant difference in overall mortality risk was observed in men (HR 0.94, 95% CI 0.88–1.0). In women the corresponding estimate was HR 0.88 (95% CI 0.82–0.94) (Table 3).

**Table 3:** Multivariable Cox proportional regression of overall mortality risk in patients with melanoma in situ (cases) and matched comparators.

Variable	All study participants overall mortality		Men overall mortality		Women overall mortality	
	Hazard ratio	95% CI	Hazard ratio	95% CI	Hazard ratio	95% CI
Cases	0.90	0.86–0.94	0.94	0.88–1.0	0.88	0.82–0.94
Comparators	1	Reference	1	Reference	1	Reference
Education						
Low	1	Reference	1	Reference	1	Reference
Middle	0.70	0.67–0.73	0.82	0.77–0.87	0.52	0.48–0.56
High	0.59	0.55–0.62	0.73	0.67–0.79	0.47	0.43–0.53
Charlson Comorbidity index						
0	1	Reference	1	Reference	1	Reference
1	2.6	2.5–2.7	2.5	2.4–2.7	2.5	2.3–2.6
2	5.0	4.8–5.3	4.70	4.4–5.0	4.4	4.0–4.7
Marital status						
Married	1	Reference	1	Reference	1	Reference
Divorced	0.93	0.88–0.97	0.95	0.89–1.0	1.0	0.95–1.1
Unmarried	0.70	0.66–0.74	0.76	0.71–0.82	0.67	0.61–0.74
Widower	2.2	2.1–2.3	2.3	2.2–2.5	2.8	2.6–2.9
Disposable income						
Below median	1	Reference	1	Reference	1	Reference
Above median	0.51	0.49–0.53	0.50	0.47–0.53	0.48	0.45–0.51

Hazard ratios and 95% confidence intervals (CI) for overall mortality during the study period. All variables are mutually adjusted.

#### Cause of death

Cardiovascular disease (ICD-10 code I05–I99) was the major cause of death in both cases (37% [841]) and comparators (40% [5117]), where chronic ischemic heart disease (ICD-10 code I25) accounted for 8.1% (184) of deaths in cases and 7.8% (1001) in comparators followed by acute myocardial infarction (ICD-10 code I21) (7.0% [160] vs 7.5% [965]).

Malignancy (CMM excluded) was the second most common cause of death in cases (27% [600]) and comparators (23% [2956]). The most frequent cancer related deaths, except CMM, in cases and comparators, respectively, were deaths in prostate cancer (ICD-10 code C61) in 3.8% (87) vs 3.1% (398), lung cancer (ICD-10 code C34) in 3.5% (80) vs 3.7% (474) and colorectal cancer (ICD-10 code C18–C20) in 3.6% (82) vs 2.8% (364). Death in CMM (ICD-10 code C43) occurred in 3.5% (79) of cases and in 0.3% (39) of the comparators. Cutaneous malignant melanoma accounted for 12% (79/679) of all cancer related deaths in cases compared to 1.3% (39/2995) in comparators.

Other common causes of deaths in MIS patients and in the matched cohort were dementia including Alzheimer's disease (ICD-10 code F03 and G30): 5.9% (134) vs 7.7% (992) and chronic obstructive pulmonary disease (ICD-10 code J44): 2.4% (54) vs 2.9% (372), respectively (Table 4).

**Table 4:** Leading causes of death in patients with melanoma in situ (cases) and matched comparators.

	Cases n (%)	Comparators n (%)	p-value
All-cause mortality	2264 (100)	12,824 (100)	
Cause of death (ICD-10 code)			
Cardiovascular disease (I05–I99)	841 (37)	5117 (40)	0.014
Chronic ischemic heart disease (I25)	184 (8.1)	1001 (7.8)	0.63
Acute myocardial infarction (I21)	160 (7.1)	965 (7.5)	0.47
Malignancy (C00–C99, C43 excluded)	600 (27)	2956 (23)	<0.001
Prostate cancer (C61)	87 (3.8)	398 (3.1)	0.076
Lung cancer (C34)	80 (3.5)	474 (3.7)	0.75
Colorectal cancer (C18–C20)	82 (3.6)	364 (2.8)	0.05
Malignant melanoma (C43)	79 (3.5)	39 (0.30)	<0.001
Dementia including Alzheimer's disease (F03 + G30)	134 (5.9)	992 (7.7)	0.003
Chronic obstructive pulmonary disease (J44)	54 (2.4)	372 (2.9)	0.20

Cause of death: Main reason for cause of death registered in the Cause of Death Register according to International Classification of Diseases, Tenth Revision (ICD -10). Number of individuals and distribution of cause of death in percent (%) among deceased individuals in each category.

## Discussion

Converging evidence shows that socio-economic factors are associated with the risk and prognosis of CMM. To the best of our knowledge, no previous study has in detail compared long-term outcomes between individuals diagnosed with MIS and the general population. By use of population-based data, we investigated whether the survival differs between patients with MIS and matched individuals free of MIS and if any such difference can be explained by socioeconomic factors and comorbidity burden. We also compared the distribution of causes of death between individuals with MIS and the background population.

We found that MIS patients had a significantly better OS compared to the comparison cohort of individuals free of MIS that remained for at least 10 years after the MIS diagnosis, a finding which is in line with the results of two earlier studies.^6^^,^^7^ Women experienced a better OS than men in both the case- and the comparison group. In multivariable analyses, a lower risk of death remained following adjustment for socioeconomic factors and comorbidity, albeit of borderline significance in men.

There were some differences between cases and comparators in the distribution of causes of death, most notably a ten-fold higher rate of melanoma-related deaths in MIS patients. Other smaller, but significant, differences among the most common causes of death were noted for cardiovascular disease, malignancies (CMM excluded) and dementia. While rates of cancer-related deaths were higher in the MIS patient group, deaths attributed to cardiovascular disease and dementia were more common in the comparison group. Of note was that a history of malignant diagnosis at baseline was significantly more common in individuals with MIS, although the prevalence of a metastatic disease was similar in both groups. This might be explained by a higher likelihood of early cancer detection in the MIS patients, possibly reflecting that high SES is associated with health awareness and health care seeking behavior.^30^, ^31^, ^32^

Strengths of the present study included the population-based setting and a virtually complete follow-up of both cases and comparators. Hence, selection bias was not an issue. Several limitations need mentioning. In the data at hand, no information was available on factors that could be associated with not only the risk of, but also the likelihood of detection and diagnosis of MIS. These include life-style factors, health care seeking behavior and medical history that may differ between cases and comparators, not fully captured by the indicators of SES and comorbidity used in the present study. Furthermore, although CCI is a widely used instrument to estimate general health status, it does not capture conditions managed in primary care such as Diabetes Mellitus type II and less severe cardiovascular conditions. Thus, the comorbidity burden is likely to have been underestimated both in cases and comparators. The differences in comorbidity and SES between cases and the comparators in our study could not fully explain the lower risk of death in individuals with MIS, at least not in women.

The rate of melanoma-related deaths was substantially higher among the MIS patients which was to be expected given the increased risk of developing a future CMM in these patients. The rate of a subsequent CMM in the MIS patients was approximately seven times higher than for the comparators, i.e. a substantially elevated risk which is in line with results in previous studies.^4^^,^^33^^,^^34^

In conclusion, we found that individuals with a diagnosis of MIS, despite being at increased risk of developing CMM, experienced a better OS compared to the background population. This finding remained following adjustment for socioeconomic factors and comorbidity. Our results have probably been affected by residual confounding, including differences in life-style factors and health seeking behavior between individuals with MIS and comparators.

Taken together our results are reassuring and should be communicated to help reduce feelings of anxiety in patients who have been diagnosed with MIS.

## Contributors

**Ylva Naeser:** Investigation, Writing - Original Draft, Project administration. **Rasmus Mikiver:** Software, Formal analysis, Data Curation, Visualization, Writing–Original Draft. **Christian Ingvar:** Conceptualization, Writing–Review & Editing, Funding acquisition. **Mats Lambe:** Conceptualization, Writing–Original Draft, Methodology, Supervision. **Gustav Ullenhag:** Conceptualization, Writing–Original Draft, Funding acquisition, Supervision.

## Data sharing statement

The study was performed by use of data from Swedish population-based registers. The Public Access to Information and Secrecy Act in Sweden prohibits us from making individual level data publicly available. Data are available by request to the steering committee of the SweMR.

## Declaration of interests

None of the authors have any competing interests to declare.
