# Supplementary material for: Survival in patients diagnosed with melanoma in situ compared to the general population. A Swedish population-based matched cohort study
Source: eClinicalMedicine. 2023 Oct 24;65:102284. doi: 10.1016/j.eclinm.2023.102284 (PMC10725068; doi:10.1016/j.eclinm.2023.102284)
Supplement: Appendix [file mmc1.pdf]

Table A1. Distribution melanoma in situ subtypes by tumor location

	Lentigo maligna (LM)	Other in situ	Total number of cases
**Tumor location**			
Trunk	482	2210	2692
Lower extremities	150	1109	1259
Upper extremities	296	992	1288
Head / neck	1813	777	2590
Palm/sole/subungual	0	55	55
Missing	21	58	79
**Total number of cases**	5201	2762	7963

Subtypes are divided into Lentigo Maligna (LM) or Other (all histological subtypes of melanoma in situ except LM).

Table A2. Multivariable (education) Cox proportional regression of overall survival in patients with melanoma in situ (cases) and matched comparators.

	All study participants<br>Overall mortality		Men<br>Overall mortality		Women<br>Overall mortality	
**Variable**	**Hazard ratio**	**95% CI**	**Hazard ratio**	**95% CI**	**Hazard ratio**	**95% CI**
Cases	0·90	0·86–0·94	0·92	0·86–0·98	0·88	0·82–0·94
Comparators	1	Reference	1	Reference	1	Reference
Education						
Low	1	Reference	1	Reference	1	Reference
Middle	0·47	0·45–0·50	0·62	0·59–0·66	0·30	0·28–0·33
High	0·35	0·33–0·37	0·48	0·44–0·52	0·24	0·22–0·27

Hazard ratios and 95% confidence intervals (CI) for overall mortality during the study period.

Table A3. Multivariable Cox (income) proportional regression of overall survival in patients with melanoma in situ (cases) and matched comparators.

	All study participants<br>Overall mortality		Men<br>Overall mortality		Women<br>Overall mortality	
**Variable**	**Hazard ratio**	**95% CI**	**Hazard ratio**	**95% CI**	**Hazard ratio**	**95% CI**
Cases	0·91	0·87–0·95	0·93	0·87–0·99	0·90	0·84–0·96
Comparators	1	Reference	1	Reference	1	Reference
Disposable income						
Below median	1	Reference	1	Reference	1	Reference
Above median	0·37	0·36–0·39	0·41	0·39–0·43	0·30	0·29–0·32

Hazard ratios and 95% confidence intervals (CI) for overall mortality during the study period.

Table A4. Multivariable Cox (comorbidity) proportional regression of overall survival in patients with melanoma in situ (cases) and matched comparators.

	All study participants<br>Overall mortality		Men<br>Overall mortality		Women<br>Overall mortality	
**Variable**	**Hazard ratio**	**95% CI**	**Hazard ratio**	**95% CI**	**Hazard ratio**	**95% CI**
Cases	0·83	0·79–0·87	0·86	0·81–0·91	0·79	0·74–0·85
Comparators	1	Reference	1	Reference	1	Reference
Charlson Comorbidity Index						
0	1	Reference	1	Reference	1	Reference
1	3·1	3·0–3.2	2·8	2·7–3·0	3·3	3·2–3·5
2	6·7	6·4–7·0	5·9	5·6–6·3	7·1	6·5–7·6

Hazard ratios and 95% confidence intervals (CI) for overall mortality during the study period.
